# Anusol for Hemorrhoids

**Published:** 1909-03

**Authors:** 


### ABSTRACTS.

### Anusol for Hemorrhoids

Coprologico-Chemical studies of hemorrhoidal cases were made by Dr. Freiherr von Oefele, of Neuenahr (*Deut. Aerzte-Zeitung,* November 15, 1908), in which he says that hemorrhoids and constipation by no means always go hand in hand; rather are they apt to exclude each other. For hemorrhoidal patients feel the passage of feces so severely, because of the anal distention caused thereby, that they persist in the use of purgatives until they attain very soft feces. Even these, however, arouse a most unpleasant sensation and a subjective feeling of constipation; but analysis of the feces will show a deficiency of solids and a large percentage of moisture. Generally speaking, a patient with hemorrhoids uses purgatives more than is good for him, as this involves waste of unutilised nutriment.

The layman is also under the impression that the uses of suppositories, such as anusol, causes softening of the feces, because defecation is rendered so much easier. This, however, is not the case, and is a factor in their favor. Thus in one case the solid contents were 5.48 per cent., and under anusol treatment rose to 7.68 per cent., later falling to 5.50 per cent. Under the subsequent use of one anusol suppository every week there was a defecation morning and evening—an improvement over the previous diarrheic condition of the patient. Generally, he found the dry contents of stools to rise under anusol treatment, but, as stated above, the relief from straining passages causes the patients to think that the stools had been softened by the suppositories. A decrease in the quantity of feces, noted by some patients, is explained by the desiccant action of the remedy and the diminution of mucosal secretion in the intestine.

During hemorrhoidal bleeding the ash contents of the excrements generally sink far below normal. Von Oefele made coprological examinations during periods without marked bleeding and therefore of feces having favorable ash contents. In these, use of anusol suppositories increased the ash contents in ten out of fourteen cases.

In hemorrhoidal subjects a large portion of the fecal ash is water-soluble, while in healthy persons only a small part is soluble in boiling distilled water. Two patients, whose stools showed especially unfavorable solubility, were treated with anusol suppositories. In one, whose soluble ash was equal to the insoluble ash, the former fell 13 per cent. in seventeen days. In the other the soluble ash contents was 45 per cent. greater than the insoluble, and after four days of anusol the former fell 14 per cent. below the latter, and finally to 67 per cent. less—a total fall to less than one-fourth of the original amount. He thus found that in hemorrhoidal patients treated with anusol the pathological excess of soluble ash decreases, and this decrease is a definite one.

The malproportion between soluble and insoluble ash is especially great in severe hemorrhoids. For in the healthy subject the chief portion of the katabolic products yielding insoluble ash is excreted by the colon, while the portion yielding soluble ash is eliminated by the kidneys. But in hemorrhoidal disease the local conditions and the fecal examination show that the affected colon is impaired in its ability of eliminating insoluble materials. Moreover, in hemorrhoids, undigested nutriment and blood and mucus are freely present; and these contain abundant soluble mineral substances and very little insoluble ones.

Before use of anusol suppositories the feces frequently contained epithelia and mucous shreds. These constituents lessened and were often superseded by mucous flakes. The healing process in the depths of the mucosæ began; there was a mobilisation of leukocytes, sometimes evidenced by their appearance in the feces; and the products of the chronic catarrh disappeared,—so that coprological examination gives results, on the basis of which the use of anusol suppositories in hemorrhoidal conditions appears commendable.

